# Human–Computer Interaction in Face Matching

**DOI:** 10.1111/cogs.12633

**Published:** 2018-06-28

**Authors:** Matthew C. Fysh, Markus Bindemann

**Affiliations:** ^1^ School of Psychology University of Kent

**Keywords:** Face matching, Face processing, Human–computer interaction, Passport control, Response bias

## Abstract

Automatic facial recognition is becoming increasingly ubiquitous in security contexts such as passport control. Currently, Automated Border Crossing (ABC) systems in the United Kingdom (UK) and the European Union (EU) require supervision from a human operator who validates correct identity judgments and overrules incorrect decisions. As the accuracy of this human–computer interaction remains unknown, this research investigated how human validation is impacted by a priori face‐matching decisions such as those made by automated face recognition software. Observers matched pairs of faces that were already labeled onscreen as depicting the same identity or two different identities. The majority of these labels provided information that was consistent with the stimuli presented, but some were also inconsistent or provided “unresolved” information. Across three experiments, accuracy consistently deteriorated on trials that were inconsistently labeled, indicating that observers’ face‐matching decisions are biased by external information such as that provided by ABCs.

## 1 Introduction

Person identification at border control relies primarily on forensic face matching. This involves a comparison between a traveler's face and their passport photograph to verify that they are the same person (i.e., an identity match) and not an impostor who is using a stolen or borrowed passport (i.e., an identity mismatch). Laboratory studies show consistently that human performance in this task can be highly error‐prone. For example, up to 20% errors arise under optimized conditions, such as when to‐be‐matched stimuli comprise high‐quality digital photographs of targets taken under even lighting with similar expressions and pose (Burton, White, & McNeill, 2010). Subsequent studies suggest that performance is further impeded by factors that are relevant to passport control. For example, accuracy on mismatch trials deteriorates considerably over a single prolonged session (Alenezi & Bindemann, 2013; Alenezi, Bindemann, Fysh, & Johnston, 2015), as well as under time pressure (Bindemann, Fysh, Cross, & Watts, 2016; Fysh & Bindemann, 2017), and when faces are viewed in the context of realistic photo ID (Bindemann & Sandford, 2011; Kemp, Towell, & Pike, 1997; McCaffery & Burton, 2016). In addition, even passport officers have been found to make a substantial number of errors when comparing same‐day face photographs (White, Kemp, Jenkins, Matheson, & Burton, 2014). Together, these findings reflect that for humans, unfamiliar‐face matching is a particularly challenging task.

Automated Border Crossing (ABC) systems present a potential solution to this problem. In the United Kingdom, for example, “Electronic Passport Gates,” or “e‐Gates,” are now installed in most major airports. These e‐Gates employ state‐of‐the‐art facial recognition algorithms that compare live travelers to a digital photograph that is stored on their passports and are unaffected by factors that impact *human* capacity for face matching, such as time pressure (Bindemann et al., 2016), time passage (Alenezi & Bindemann, 2013; Alenezi et al., 2015), and sleep deprivation (Beattie, Walsh, McLaren, Biello, & White, 2016). These benefits are corroborated by studies in which face recognition algorithms have achieved perfect or near‐perfect performance in benchmark tests (see, e.g., Phillips et al., 2010; see also Jenkins & Burton, 2008).

Despite these advantages, however, it remains difficult to establish the accuracy of automatic facial recognition systems in applied contexts. For example, algorithms outperform human observers in tests that are considered to be of easy and moderate difficulty (O'Toole, An, Dunlop, Natu, & Phillips, 2012; O'Toole et al., 2007). However, under more challenging conditions that more closely approximate passport control, such as when to‐be‐compared stimuli are photographed on different days, these algorithms perform comparably to some observers (O'Toole et al., 2012; Phillips & O'Toole, 2014) and are surpassed by expert matchers (White, Phillips, Hahn, Hill, & O'Toole, 2015). Some studies have also reported instances where face recognition algorithms failed to score even a single hit in matching tasks, while humans were well above chance (Rice, Phillips, Natu, An, & O'Toole, 2013). Together, these findings indicate that algorithms are not yet fully capable of supplanting humans at border control.

Currently, e‐Gates function under the supervision of human operators, who monitor the identity verification process via a computer interface. This interface indicates the extent to which the person in the e‐Gate booth matches his or her passport photograph, as well as notifies the operator when a potential biometric mismatch is present (for an example of this interface, see Secunet, n.d.). A critical responsibility of the human operators is therefore to manage exceptions such as when the system cannot fully resolve a traveler with his or her passport photograph, as well as to prevent the system from incorrectly accepting a mismatching identity or incorrectly rejecting a genuine match (FRONTEX, 2015a,2015b; Graves et al., 2011; Secunet, n.d.). Such errors are projected to occur only rarely, with the false acceptance of impostors estimated to occur on 0.1% of trials, and the false rejection of identity matches on 5%–10% of trials (FRONTEX, 2015b). These error rates are not represented in applied contexts, where e‐Gates have been reported to reject high volumes of identity matches (Independent Chief Inspector of Borders and Immigration, 2014; Watt, 2016), and to falsely accept some egregious mismatches, such as men as women (http://www.bbc.co.uk/news/uk-england-manchester-12482156; Independent Chief Inspector of Borders and Immigration, 2011). These surprising errors indicate that the interaction between humans and e‐Gates is crucial for the accuracy of person identification at passport control. However, the accuracy of this human–computer interaction is currently unknown.

So far, only limited research has explored this issue. In one study, the face‐matching decisions of humans and algorithms were aggregated together, resulting in near‐perfect performance (O'Toole, Abdi, Jiang, & Phillips, 2007). However, the judgments of human observers in this study were independent of those made by algorithms, which differs from applied settings, where human operators instead validate a priori judgments by e‐Gates. A more recent study investigated the performance of facial review staff, who use state‐of‐the‐art face recognition algorithms to process new passport applications (White, Dunn, Schmid, & Kemp, 2015). In this task, the algorithm compares the face of a passport applicant across a database of existing passport holders to prevent fraudulent applications from being processed. The algorithm returns eight candidates who most closely resemble the applicant, which are then studied by the human operator to ensure that the applicant's photograph does not match that of any existing passport holders. Importantly, the researchers found that the accuracy of facial review staff actually limited the success of the algorithm, which could reliably return a matching identity from a database of over a million candidates.

This research suggests that person identification accuracy might not benefit from this human–computer interaction. However, crucial differences between the role of facial review staff and human operators at passport control make it difficult to generalize White, Dunn, et al.'s (2015) findings to the latter context. For example, facial review staff are required to check a single candidate image against eight highly similar face photographs, to safeguard against fraudulent passport applications. In contrast, the operators of e‐Gates at passport control perform a secondary comparison on *pairs* of faces, for which an identification has already been provided by the system. Although this task may bear some conceptual similarity to the paradigm explored by White, Dunn, et al. (2015), important theoretical differences exist between these tasks, making it difficult to draw firm conclusions about human performance in one task from the other. For example, the role of human operators of e‐Gates rests on the critical assumption that human operators’ identification decisions occur independent of the system output. Thus, human operators should overrule the system when the algorithm falsely rejects an identity match, or falsely accepts an identity mismatch. If this is correct, then the identity decisions of humans and algorithms should align when the system has made a correct judgment, but also diverge when the system is incorrect.

Psychological research provides mixed evidence for this assumption. On one hand, attentional load paradigms demonstrate that face identity processing is disrupted when face targets are flanked by semantically relevant words (Bindemann, Burton, & Jenkins, 2005). This interference effect is characterized by longer response times and reduced accuracy when distractors provide information that is incongruent, as compared to congruent, with the target identity (Bindemann et al., 2005). In addition, identification judgments for faces can be influenced by some simple manipulations relevant to forensic face matching. For example, embedding face photographs within passport frames increases false acceptance of identity mismatches between a passport photo and its bearer (McCaffery & Burton, 2016). These findings suggest that human face‐matching decisions may also be influenced by identification judgments provided by e‐Gates, whereby the identity decisions by the system may reduce the decision accuracy of human observers when the system is incorrect.

On the other hand, it is conceivable also that decisions by human operators are relatively unaffected by e‐Gate judgments. Faces capture and retain attention more effectively than other stimulus categories (see, e.g., Bindemann, Burton, Hooge, Jenkins, & de Haan, 2005; Bindemann, Burton, Langton, Schweinberger, & Doherty, 2007; Langton, Law, Burton, & Schweinberger, 2008; Theeuwes & Van der Stigchel, 2006). Moreover, task‐irrelevant face distractors tend to interfere with the processing of attended non‐face targets more strongly than task‐irrelevant non‐face stimuli with attended faces (Bindemann et al., 2005). It is therefore possible in human–computer interaction scenarios that the facial information within to‐be‐matched faces exerts a greater influence on human decision‐making than information from computer algorithms. Consequently, the face‐matching decisions of human operators might also be unaffected by e‐Gate judgments.

Considered together, it remains difficult from the current evidence to predict whether human operators can make identification decisions independently of the output of e‐Gate systems. Consequently, it is unresolved whether identity verification accuracy at passport control is improved by human–computer interaction or, paradoxically, reduced by it. This question is explored in this study. Across three experiments, observers matched pairs of faces that were labeled as depicting the “same” person, “different” individuals, or that were “unresolved.” Labels that provided a *same* or *different* resolution were generally consistent with the faces shown. However, a small percentage of these also provided inconsistent information. In these cases, match trials were incorrectly labeled as different individuals, and mismatch trials were labeled as depicting the same person. Unresolved trials were chosen as an analogy to the exceptions at e‐Gates when a traveler cannot be matched by the algorithm, and thus *must* be processed by the human operator. Experiment 1 first sought to determine whether observers can accurately match faces while instructed to ignore the information provided by these trial labels. Subsequent experiments investigated how responses are further affected when observers instead attempt to verify the accuracy of labels (Experiment 2), and whether high compliance with the labels facilitates accuracy gains when these provide consistent information, at the cost of performance on trials for which the labels are misleading (Experiment 3).

## 2 Experiment 1

In this experiment, observers matched pairs of faces that were labeled onscreen as belonging to the “same” person, “different” individuals, or as “unresolved” identity pairings. These labels were employed as an analogy to human–computer interaction at passport control, where human operators supervise e‐Gates judgments for each identity, to prevent the false acceptance of impostors by the system. For this to be effective, human operators should be making identifications that are independent of the algorithm judgments and can therefore overrule erroneous e‐Gate decisions. The aim of this experiment was to investigate whether observers can match faces without being influenced by onscreen identity judgments.

To investigate this, we informed observers that most of the onscreen labels provided a correct identification for each trial, but that some were also inaccurate, and that they should therefore ignore the labels when deciding whether faces depicted the same person or different individuals. To understand this within the context of passport control, the stimuli that were employed in this study portrayed considerable within‐person variability (see Jenkins, White, Van Montfort, & Burton, 2011; Megreya, Sandford, & Burton, 2013), and mismatches occurred infrequently (Bindemann, Avetisyan, & Blackwell, 2010; Papesh & Goldinger, 2014). Furthermore, the proportion of inconsistently labeled and unresolved trials was lower than the proportion of trials with consistent labels, given that the algorithms employed at passport control are projected to be highly accurate, and thus incorrect identifications should occur only rarely (FRONTEX, 2015b). This design should indicate whether human performance in face matching is influenced by onscreen trial information, as an analogy to human–computer interaction at passport control.

### 2.1 Methods

#### 2.1.1 Participants

Thirty undergraduates (7 males, 23 females) with a mean age of 22.1 years (*SD* = 7.3) studying at the University of Kent participated in this research in exchange for course credit. All reported normal (or corrected‐to‐normal) vision. This study was approved by the Ethics Committee of the School of Psychology at the University of Kent and was conducted in accordance with the ethical guidelines of the British Psychological Association.

#### 2.1.2 Stimuli

The stimuli in this study comprised 210 pairs of faces extracted from the Kent University Face Database (KUFD; see Fysh & Bindemann, 2018). Of these, 15 were mismatching identities, and the remaining 195 were identity matches. One photo in each pair consisted of a controlled image, in which targets were photographed against a plain white background under even lighting and whilst bearing a neutral expression. These photographs were cropped to depict the target's head and shoulders, and they were scaled to a size of 283 × 332 pixels at a resolution of 72 ppi, before being placed on the right‐hand side of a plain white canvas. The second image consisted of a student ID photograph that was retrieved with permission from the University of Kent's online Student Data System. These images were unconstrained in target expression, pose, and lighting; therefore, contribute an important source of variability to each stimulus pair. To preserve resolution, these photographs were scaled to a size of 142 × 192 pixels at a resolution of 72 ppi, and were presented to the left of the controlled images. Mismatching pairs were created by pairing identities that were visually similar in terms of hair color, face shape, and eyebrow shape.

**Figure 1:**
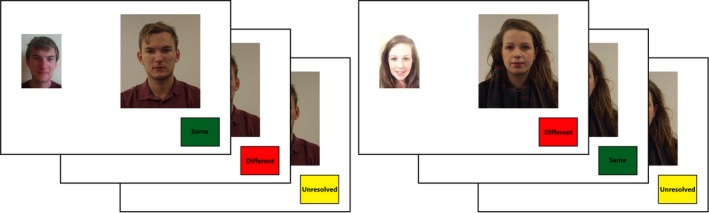
Example stimuli used across Experiments 1–3. The left pair depicts two images of the same person with a consistent (i.e., correct), inconsistent (incorrect), and unresolved trial label. The right pair depicts two different individuals with a consistent (correct), inconsistent (incorrect), and unresolved trial label.

Next, three versions of each face‐pair stimulus were created, featuring a trial label in the bottom right corner of the canvas that displayed the message “same,” “different,” or “unresolved” (see Fig. 1). Each observer viewed one version of each stimulus. However, to ensure that each identity pair was presented alongside a consistent, inconsistent, and unresolved label with equal frequency across observers, these were counterbalanced over 15 versions of the task. Each trial label measured 137 × 101 pixels. To provide a closer analogy to the interface used by human operators of ABC systems (see Secunet, n.d.), these labels were also colored green, red, or yellow, corresponding to whether the label was “same,” “different,” or “unresolved,” respectively.

#### 2.1.3 Procedure

This experiment was run using *PsychoPy* software (Peirce, 2007). Trials were divided evenly over three blocks of 70 face pairs (65 matches, 5 mismatches), which proceeded without any breaks. At the beginning of the task, observers were instructed that an identity judgment had already been supplied for each face pair, and that while the majority of these would be correct, some would be inaccurate. It was therefore important that observers ignored the trial labels when deciding whether the faces onscreen portrayed the same person or different individuals.

Each trial was preceded by a 1‐second fixation cross. This was then replaced with a stimulus pair that was labeled onscreen as “same,” “different,” or “unresolved.” The majority of the trial labels (60%) provided consistent information about the face pair. However, 20% of the labels were also inconsistent, in that they displayed the incorrect solution to the onscreen faces. The remaining 20% of trial labels were *unresolved*, such that observers were required to independently decide whether two faces depicted the same person or two different individuals. Thus, for the 65 identity matches in a block of 70 trials, 39 were presented with a consistent identification label, 13 with an inconsistent label, and another 13 with an unresolved label. Equally, for the five identity mismatches in each block, three were presented with a consistent identification label, one with an inconsistent label, and another with an unresolved label.

### 2.2 Results

**Figure 2:**
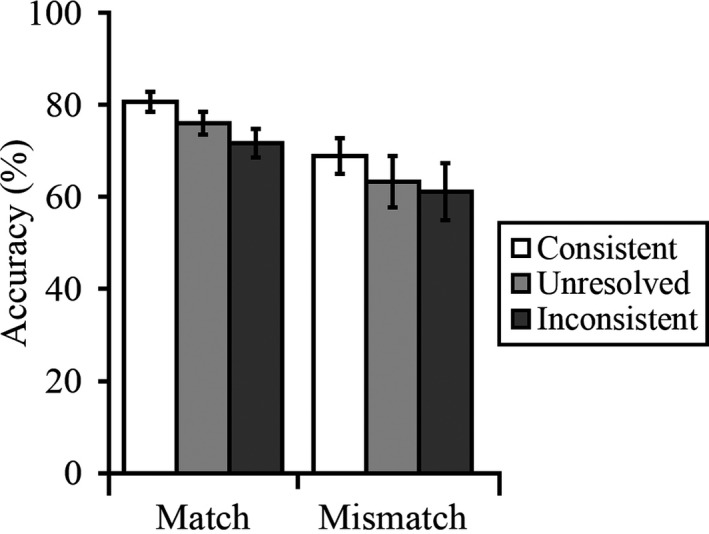
Percentage accuracy scores for Experiment 1. Error bars represent the standard error of the mean.

The percentage accuracy data were calculated for all conditions. The cross‐subject means of these data are provided in Fig. 2. To maximize the number of data points in this analysis, the accuracy data were collapsed across the three blocks of the experiment. A 2 (trial type: match vs. mismatch) × 3 (trial label: consistent, unresolved, inconsistent) within‐subjects Analysis of Variance (ANOVA) was conducted, which did not reveal a main effect of trial type, *F*(1, 29) = 4.00, *p *=* *.06, η_p_^2^ = 0.12, or an interaction, *F*(2, 58) = 0.06, *p *=* *.94, η_p_^2^ = 0.00. However, a main effect of trial label was found, *F*(2, 58) = 4.20, *p *<* *.05, η_p_^2^ = 0.13. To interpret this effect, the percentage accuracy data were collapsed across match and mismatch trials and were then compared for consistent, inconsistent, and unresolved trial labels via a series of paired‐sample *t* tests (with *alpha* corrected at *p *<* *.017 [i.e., 0.05/3] for multiple comparisons). This analysis did not find significant differences between unresolved and consistent trials, *t*(29) = 1.97, *p *=* *.06, and between unresolved and inconsistent trials, *t*(29) = 1.01, *p *=* *.32. However, accuracy on consistent trials was higher than on inconsistent trials, *t*(29) = 2.93, *p *<* *.01. This demonstrates that trial labels influenced observers’ face‐matching decisions despite the instruction to ignore these.

See Supplemental Material for additional analysis of *d’*,* criterion*, and response times for all experiments reported here.

### 2.3 Discussion

In this experiment, observers matched pairs of faces that were labeled onscreen as depicting *same* or *different* identities or as *unresolved* identifications. Observers were informed at the start of the task that the majority of the labels provided correct information, but that some were also inaccurate, and that it was therefore important to ignore the labels when matching faces. Despite this instruction, the labels clearly affected face‐matching performance. Accuracy for consistently labeled match and mismatch trials was 81% and 69%, respectively, but declined to 72% and 61% when the labels provided inconsistent identity information for these trials. In addition, performance for unresolved match and mismatch trials fell in between these conditions and was 76% and 63%, respectively.

These findings indicate that a priori external identity judgments, such as same‐ and different‐identity labels, interfere with observers’ face‐matching decisions even when attempting to ignore such information. This converges with work showing that observers’ face‐matching decisions can be compromised when led to believe that mismatching faces depict the same person (Menon, White, & Kemp, 2015), as well as with research demonstrating that face identity processing can be disrupted when flanked by semantically relevant distractor words (Bindemann et al., 2005).

This experiment indicates that human operators may struggle to ignore the information provided by e‐Gate systems when processing travelers. However, it is conceivable that in such operational settings, operators of e‐Gates may instead attempt to evaluate the accuracy of each system identification, rather than ignoring these judgments per se. This strategy makes some sense when considering that e‐Gates are projected to be highly accurate, and should, in theory, make very few errors (FRONTEX, 2015b). By contrast, human face‐matching performance deteriorates due to the repetitive nature of the task (Alenezi & Bindemann, 2013; Alenezi et al., 2015), and when under time pressure (Bindemann et al., 2016; Fysh & Bindemann, 2017; Wirth & Carbon, 2017). Consequently, human–computer interaction at passport control may be more effective if human operators instead seek to verify e‐Gate judgments, rather than ignore them outright. We explore this possibility in Experiment 2.

## 3 Experiment 2

The previous experiment suggested that onscreen identity judgments interfere with face identification decisions even when observers attempt to ignore such information. However, a more effective strategy employed by human operators at passport control may be to assess the accuracy of the e‐Gate decision for each traveler, to verify that a correct decision has been made. In practice, utilizing the high accuracy of e‐Gates in this way should lead to fewer errors (FRONTEX, 2015b). However, it remains unclear as to whether observers could reliably overrule e‐Gate errors using this strategy. To explore this question in the current experiment, we repeated the procedure used in Experiment 1 but amended the instructions to encourage use of the onscreen labels. Observers were instructed that while most trial labels were correct, some were also inaccurate, and so it was important to check each pair of faces carefully before submitting the final decision.

This design should indicate whether trial labels also interfere with observers’ face‐matching decisions when explicitly evaluating the accuracy of these judgments on each trial, rather than ignoring them. Current evidence shows that observers are likely to conform to algorithm judgments in decision‐making tasks when the outcome of a given trial is ambiguous (Weger, Loughnan, Sharma, & Gonidis, 2015). In addition, although an attentional bias exists for faces, observers also utilize task‐relevant non‐face objects in perceptual tasks when it is advantageous to do so (Bindemann et al., 2007). Extending these findings to the current experiment, we expect to observe further interference from the trial labels when observers match pairs of faces. This interference effect should be characterized by high accuracy on trials for which the labels provide consistent information, but may also coincide with poorer accuracy on trials that are inconsistently labeled.

### 3.1 Method

#### 3.1.1 Participants, stimuli, and procedure

Thirty undergraduates studying at the University of Kent (11 males, 19 females) with a mean age of 20 years (*SD* = 3.8) participated in this research in exchange for course credit or a small fee. All reported normal (or corrected‐to‐normal) vision, and none had participated in Experiment 1.

The stimuli and procedure in this experiment were identical to the previous experiment, except for the following change. Instead of instructing observers to ignore the trial labels as in Experiment 1, participants were informed that while the majority of trial labels were correct, some would also be inaccurate, and that it was therefore important to check each identity pair carefully before submitting the final decision.

### 3.2 Results

**Figure 3:**
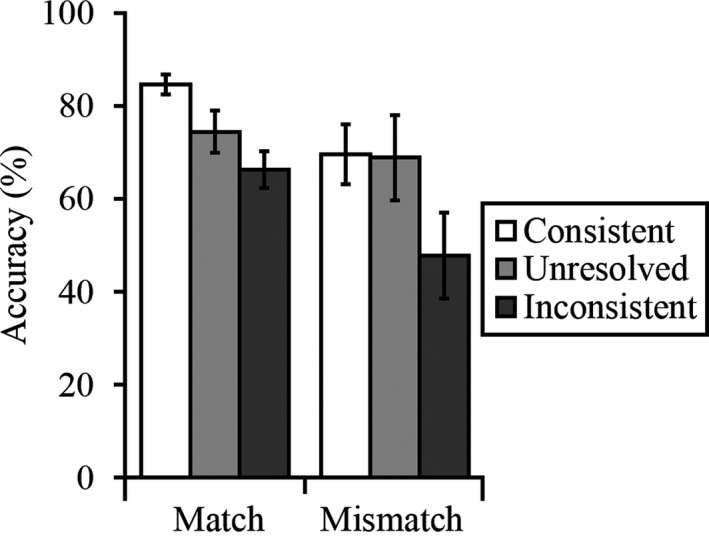
Percentage accuracy scores for Experiment 2. Error bars represent the standard error of the mean.

The cross‐subject mean percentage accuracy scores for consistent, inconsistent, and unresolved match and mismatch trials were analyzed (see Fig. 3). A 2 (trial type: match vs. mismatch) × 3 (trial label: consistent, unresolved, inconsistent) within‐subjects ANOVA revealed a main effect of trial type, *F*(1, 29) = 5.25, *p *<* *.05, ƞ_p_^2^ = 0.15, a main effect of trial label, *F*(2, 58) = 12.14, *p *<* *.001, ƞ_p_^2^ = 0.30, and an interaction between these factors, *F*(2, 58) = 3.21, *p *<* *.05, ƞ_p_^2^ = 0.10.

Analysis of simple main effects showed that accuracy was superior on match compared to mismatch trials with consistent labels, *F*(1, 29) = 8.62, *p *<* *.01, ƞ_p_^2^ = 0.23, and inconsistent labels, *F*(1, 29) = 6.44, *p *<* *.05, ƞ_p_^2^ = 0.18, but comparable with unresolved labels, *F*(1, 29) = 0.68, *p *=* *.42, ƞ_p_^2^ = 0.02. More important, simple main effects analysis revealed also that performance on both match, *F*(2, 28) = 6.32, *p *<* *.01, ƞ_p_^2^ = 0.31, and mismatch trials was affected by the trial labels, *F*(2, 28) = 6.66, *p *<* *.01, ƞ_p_^2^ = 0.32.

For the simple main effect of match trials, paired‐sample *t* tests (with *alpha* corrected at *p *<* *.017 for multiple comparisons) showed that accuracy was higher when the labels were consistent versus when they were unresolved or inconsistent, *t*(29) = 3.36, *p *<* *.01 and *t*(29) = 3.59, *p *<* *.01, respectively. In addition, accuracy on unresolved match trials was also higher than when these pairs were labeled inconsistently, *t*(29) = 2.85, *p *<* *.01. Likewise, corrected paired‐sample *t* tests were performed to explore the effect of the trial labels on mismatch trials. These revealed lower accuracy on inconsistently labeled trials compared to when the labels were consistent, *t*(29) = 3.45, *p *<* *.01, and unresolved, *t*(29) = 3.36, *p *<* *.01. However, mismatch accuracy was comparable between consistent and unresolved trials, *t*(29) = 0.15, *p *=* *.89.

### 3.3 Discussion

This experiment showed that when verifying the accuracy of trial labels, observers’ face‐matching decisions are influenced by the information that these provide. Performance was considerably more accurate when these labels provided information that was consistent with the identities of the depicted face pairs. For example, accuracy on mismatch trials deteriorated from 70% when these were labeled as depicting different identities (i.e., with a consistent label) to 48% when faces were labeled as belonging to the same person (inconsistent label). Similarly, performance on match trials deteriorated from 85% when the labels were consistent, to 66% when these indicated that two faces depicted different individuals. For trials that were labeled as unresolved, accuracy was similar between match and mismatch trials, at 74% and 69%, respectively.

Considered alongside Experiment 1, these findings show that the trial labels interfere with face‐matching decisions not only when observers attempt to ignore the judgments that are presented, but also when observers specifically attend to these throughout the task. This is particularly troubling when considering the observed interaction, which demonstrated a more pronounced effect of inconsistent labels on mismatch trials compared to match trials. Translated to applied settings, this suggests that human operators are less likely to detect the false acceptance of impostors by e‐Gates than the false rejection of identity matches.

Although the trial labels influenced responses in this task, accuracy on consistently labeled trials was lower than would be expected (i.e., 100%) if observers were resolutely following the labels. This indicates that observers were reluctant to conform to the judgments provided by the trial labels, and that the facial information in stimuli were exerting a stronger influence on identification decisions. One possible explanation for this is that observers may have encountered misleading labels early on in the task and were therefore less willing to utilize the information presented. This makes it difficult to apply these findings to passport control, where human operators are likely to be more trusting of the algorithms’ decisions in such settings, given that these systems are expected to be highly accurate (FRONTEX, 2015b). To encapsulate this, we ran a final experiment, in which we sought to encourage compliance with the trial labels through the administration of feedback in Block 1, and replaced all inconsistent labels in Blocks 1 and 2 to provide consistent information.

## 4 Experiment 3

In the previous experiment, accuracy deteriorated by around 20% on inconsistently labeled match and mismatch trials. However, observers also rejected nearly a quarter of consistent labels. Converging with research showing that human conformity to computer judgments is modulated by the ambiguity of a given trial (Weger et al., 2015), it is possible that conformity to the trial labels was reduced by a number of inconsistently labeled, low‐ambiguity trials at the start of the task.

To investigate this possibility, all inconsistent labels in Blocks 1 and 2 of Experiment 3 were replaced to provide consistent information, so that observers did not encounter any misleading trial labels until the final block of the task. To further encourage compliance with the trial labels, feedback was also administered in Block 1 while stimuli were still onscreen. Considering research showing that face‐matching performance benefits reliably from feedback (Alenezi & Bindemann, 2013; White, Kemp, Jenkins, & Burton, 2014), observers should display high compliance with trial labels over the course of Block 1, which should be maintained in Block 2, given that this block also did not feature any inconsistent trial labels. In the final block, this should coincide with high accuracy on trials for which the labels are consistent but result in an even greater number of errors on inconsistent trials. Moreover, we expect that these errors will be exaggerated on inconsistent mismatch trials, given that these occurred less frequently than inconsistent match trials.

### 4.1 Method

#### 4.1.1 Participants, stimuli, and procedure

Thirty undergraduates studying at the University of Kent (8 males, 22 females) with a mean age of 19.6 years (*SD* = 1.8) participated in this study in exchange for course credit or a small fee. None of these had participated in the previous experiments, and all reported normal (or corrected‐to‐normal) vision.

The stimuli and procedure used in this experiment were identical to that of the previous experiment, except for the following changes. All inconsistent labels in Blocks 1 and 2 were replaced to provide consistent information, while the frequency of unresolved match and mismatch trials remained unchanged. In addition, onscreen feedback was provided following each response in Block 1, while the stimuli and labels were still onscreen, and consisted of “Correct/Incorrect! These faces show the SAME person/two DIFFERENT individuals!” This feedback was withdrawn in Block 2, and Block 3 was identical to the third block in Experiments 1 and 2.

### 4.2 Results

#### 4.2.1 Accuracy for Blocks 1 and 2

**Figure 4:**
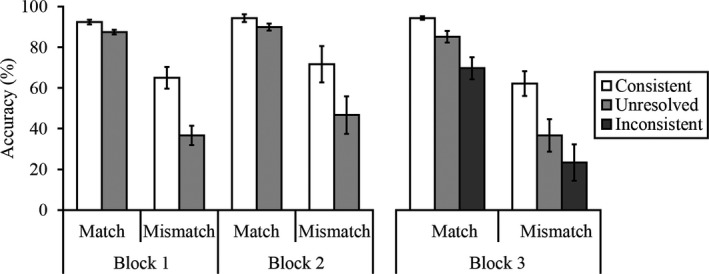
Percentage accuracy scores for Experiment 3. Error bars represent the standard error of the mean.

First, we assessed performance for Block 1 and 2 (see Fig. 4). A 2 (trial type: match vs. mismatch) × 2 (block: Block 1 vs. Block 2) × 2 (trial label: consistent vs. unresolved) within‐subjects ANOVA did not reveal an effect of block, *F*(1, 29) = 2.16, *p *=* *.15, ƞ_p_^2^ = 0.07, or interactions of block and trial label or trial type, both *Fs* ≤ 0.67, both *ps* ≥ .42, both ƞ_p_^2^ ≤ 0.02. The three‐way interaction was also not significant, *F*(1, 29) = 0.04, *p *=* *.85, η_p_^2^ = 0.00. However, an interaction of trial type and trial label was found, *F*(1, 29) = 10.20, *p *<* *.01, η_p_^2^ = 0.26. Simple main effects analysis revealed that accuracy on match trials was higher than on mismatch trials for consistent trials, *F*(1, 29) = 47.06, *p *<* *.001, η_p_^2^ = 0.62, and unresolved trials, *F*(1, 29) = 44.82, *p *<* *.001, η_p_^2^ = 0.61. More important, simple main effects of trial label were found for match, *F*(1, 29) = 6.27, *p *<* *.05, η_p_^2^ = 0.18, and mismatch trials, *F*(1, 29) = 14.48, *p *<* *.01, η_p_^2^ = 0.33, due to higher accuracy on consistent versus unresolved trials. This indicates that observers’ decisions were guided by the trial labels.

#### 4.2.2 Accuracy for Block 3

The data of main interest concerned the extent to which observers were able to detect misleading trial labels in Block 3 (see Fig. 4). A 2 (trial type: match vs. mismatch) × 3 (trial label: consistent, unresolved, inconsistent) within‐subjects anova of this data did not reveal an interaction, *F*(2, 58) = 1.60, *p *=* *.21, η_p_^2^ = .05, but revealed a main effect of trial type, *F*(1, 29) = 46.93, *p *<* *.001, ƞ_p_^2^ = 0.62, due to higher accuracy on match compared to mismatch trials. A main effect of trial label was also found, *F*(2, 58) = 13.17, *p *<* *.001, ƞ_p_^2^ = 0.31. Paired‐sample *t* tests (with *alpha* corrected at *p *<* *.017) revealed that this was due to higher accuracy on consistently labeled trials compared to unresolved trials, *t*(29) = 3.07, *p *<* *.01, and compared to trials for which the labels were inconsistent, *t*(29) = 5.09, *p *<* *.001. However, accuracy on unresolved trials was comparable to inconsistent trials, *t*(29) = 2.17, *p *=* *.04. These findings reflect that observers found it difficult to overrule inconsistent trial labels in this block.

**Figure 5:**
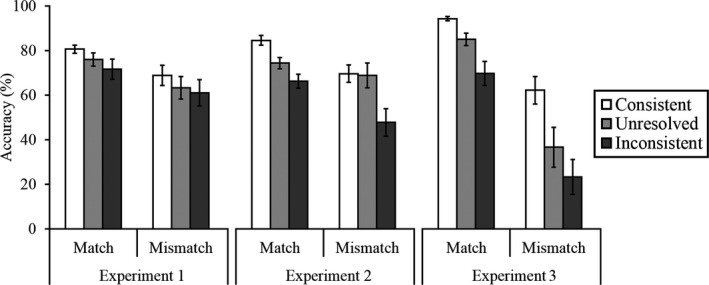
Percentage accuracy scores collapsed across Blocks 1–3 for Experiments 1 and 2, and for Block 3 of Experiment 3. Error bars represent the standard error of the mean.

In a final step of the analysis, we sought to confirm whether Experiment 3, which was designed to induce stronger compliance with the trial labels, was more likely to produce errors than Experiments 1 and 2 (see Fig. 5). To achieve this, a cross‐experiment comparison was performed on the percentage accuracy data for Experiments 1 and 2, collapsed across Blocks 1‐3, and accuracy in Block 3 of Experiment 3.^1^ This revealed main effects of experiment, *F*(2, 87) = 5.19, *p *<* *.01, η_p_^2^ = 0.12, trial type, *F*(1, 87) = 42.93, *p *<* *.001, η_p_^2^ = 0.33, and of trial label, *F*(2, 174) = 28.10, *p *<* *.001, η_p_^2^ = 0.24. These were qualified by significant interactions between experiment and trial type, *F*(2, 87) = 8.59, *p *<* *.001, η_p_^2^ = 0.17, and between experiment and trial label, *F*(4, 174) = 3.53, *p *<* *.01, η_p_^2^ = 0.08. However, trial type and trial label did not interact, *F*(2, 174) = 0.84, *p *=* *.43, η_p_^2^ = 0.01, and the three‐way interaction was not significant, *F*(4, 174) = 1.86, *p *=* *.12, η_p_^2^ = 0.04.

Analysis of simple main effects for the interaction of experiment and trial type revealed that accuracy on both match and mismatch trials varied between experiments, *F*(2, 87) = 3.18, *p *<* *.05, η_p_^2^ = 0.07 and *F*(2, 87) = 8.91, *p *<* *.001, η_p_^2^ = 0.17, respectively. To interpret this, the percentage accuracy data for match and mismatch trials were collapsed across trial label categories for each experiment and were then compared between experiments via a series of independent‐sample *t* tests (with *alpha* corrected at *p *<* *.017). These revealed comparable accuracy on match trials between all experiments following the Bonferroni adjustment, all *t*s ≤ 2.23, all *p*s ≥ .03. For mismatch trials, accuracy was also comparable between Experiments 1 and 2, *t*(58) = 0.41, *p = *.69, but was higher in Experiment 1 compared to Experiment 3, *t*(58) = 3.64, *p *<* *.01, and in Experiment 2 compared to Experiment 3, *t*(58) = 3.42, *p *<* *.01.

More important, simple main effects analysis for the interaction of experiment and trial label revealed comparable accuracy across Experiments 1–3 on consistently labeled trials, *F*(2, 87) = 0.55, *p *=* *.58, η_p_^2^ = 0.01, as well as on trials that were unresolved, *F*(2, 87) = 2.86, *p *=* *0.06, η_p_^2^ = 0.06. For inconsistent trials, however, accuracy varied between experiments, *F*(2, 87) = 6.34, *p *<* *.01, η_p_^2^ = 0.13. To interpret this effect, the percentage accuracy data were collapsed across inconsistently labeled match and mismatch trials for each experiment, and compared across experiments via a series of independent‐sample *t* tests (with *alpha* corrected at *p *<* *.017). These revealed comparable accuracy between Experiments 1 and 2, *t*(58) = 1.99, *p *=* *.05, and Experiments 2 and 3, *t*(58) = 1.68, *p *=* *.10. However, accuracy was significantly poorer on inconsistently labeled trials in Experiment 3 compared to Experiment 1, *t*(58) = 3.50, *p *<* *.01. This suggests that high trust in the trial labels exerted a greater effect on accuracy when these provided information that was inconsistent with the trial type, compared to when observers were attempting to ignore the information that these provided.

### 4.3 Discussion

This experiment provides further evidence that onscreen trial labels interfere with face matching judgments. Accuracy was near‐ceiling for consistent match trials in Block 1, indicating high compliance with the most frequent trial type. Accuracy was also similar between Blocks 1 and 2, suggesting that observers remained compliant with the labels after the feedback was withdrawn. Importantly, however, accuracy on inconsistently labeled trials was lower than on consistently labeled trials in Block 3. Moreover, the compliance manipulation of this experiment produced a greater number of errors on inconsistently labeled trials in the final block in comparison with Experiment 1, in which observers were instructed to ignore the labels completely, whereas accuracy for Experiment 2, with its emphasis on verifying trial label information, fell in‐between. In addition, a deficit in mismatch accuracy was also observed in the third block of this experiment, compared to Experiments 1 and 2. Together, these results suggest that the administration of feedback further increases observers’ reliance on the trial labels. If human operators at passport control come to rely on e‐Gates to a similar extent, then human–computer interactions would lead to increased failure to detect instances where algorithms falsely reject identity matches and, more critically, falsely accept identity mismatches.

## 5 General discussion

This study investigated face‐matching accuracy while onscreen trial labels provided consistent, inconsistent, or unresolved information about to‐be‐matched faces. Observers were informed that most of these labels presented the correct response, but that some would also be inaccurate as well as unresolved. In each experiment, the trial labels impacted performance, with accuracy deteriorating considerably between consistent and inconsistent trial labels. In Experiment 1, observers were instructed to ignore the trial labels. The purpose of this was to determine whether it was possible for observers to independently compare faces despite the onscreen presence of these labels. On match trials, accuracy deteriorated by 9% between consistent and inconsistent trial labels, and by 8% on mismatch trials. This indicates that interference from the trial labels occurs even when observers attempt to ignore the information provided by the trial labels.

In Experiment 2, we instructed observers to check the outcome of each trial label carefully before submitting a final identification decision. This resulted in 18% and 22% more errors on inconsistent match and mismatch trials, respectively, compared to when these were consistent. However, even though observers were aware that the majority of the labels provided the correct solution to the trials, accuracy was nonetheless below ceiling on match (85%) and mismatch (70%) trials, indicating that observers were reluctant to trust the trial labels even when these provided accurate information.

The final experiment sought to encourage compliance with the trial labels, by providing trial‐by‐trial feedback in Block 1, and omitting any inconsistent trial labels until Block 3. As a result of this manipulation, accuracy deteriorated between consistent and inconsistent trial labels by 25% and 39% on match and mismatch trials, respectively. Considered together, these experiments demonstrate clearly that human decisions in face matching are influenced by onscreen identifications, resulting in accuracy gains when this information is correct, but also increases in error when this is misleading.

These findings converge with a recent study, which found that human performance in face matching curtailed the accuracy of algorithms when processing passport applications (White et al., 2015). This study suggests that human–computer interaction at passport control is also error‐prone. Paradoxically, however, the reported experiments indicate that the commission of errors by algorithms facilitates errors in humans, given that observers were more likely to accept a mismatch, and reject an identity match, if these were labeled as depicting the same person or different individuals, respectively. This finding aligns with evidence that facial identification processes are guided by information from trustworthy sources, such as experimenters, even when inaccurate (see, e.g., Johansson, Hall, Sikström, & Olsson, 2005; Menon et al., 2015; Sagana, Sauerland, & Merckelbach, 2016; Sauerland et al., 2016). In addition, human operators are typically expected to monitor up to seven e‐Gates concurrently (FRONTEX, 2015a). This raises further concerns when considering that in laboratory settings, face matching suffers considerably when observers are expected to process more than one concurrent identity (see, Bindemann, Sandford, Gillatt, Avetisyan, & Megreya, 2012; Megreya & Burton, 2006). As a consequence, it is possible that the task of human operators is substantially more challenging still than the current results suggest.

It is worth noting that across all experiments, observers refrained from complying fully with the trial labels. For example, although observers erroneously accepted many inconsistent trial labels, performance on consistent trials was also repeatedly below ceiling, whereby observers incorrectly overruled the labels. This reluctance to trust the labels is perhaps surprising, given that these generally provided consistent information. However, the use of feedback and the omission of inconsistent labels in Blocks 1 and 2 of Experiment 3 increased compliance with the onscreen identifications. This reduced accuracy on inconsistently labeled trials in Block 3 of Experiment 3 compared to in Experiment 1, suggesting that high trust in the labels reduced the probability that observers would detect inconsistent labels, relative to when attempting to ignore these judgments. This raises the possibility that in this block, observers were strategically attending to the trial labels when processing stimuli, rather than utilizing the information conveyed by the facial stimuli. This converges with additional evidence showing that although an attentional bias exists for faces, observers can also endogenously shift their attention to non‐face stimuli that are task‐relevant, when this confers an advantage to the task at hand (Bindemann et al., 2007).

Across Experiments 1–3, performance on unresolved trials ranged from 74%–88% on match trials, and 40%–69% on mismatch trials. This resonates with the consistent finding that face matching is also error‐prone when an a priori judgment is not provided (e.g., Burton et al., 2010; Fysh & Bindemann, 2018). This raises additional concerns surrounding the identification accuracy of human operators of e‐Gates when the system cannot adequately resolve a person with their passport photograph (FRONTEX, 2015a). However, accuracy on these unresolved trials was generally superior to when the trial labels provided inconsistent information, reflecting that it is more challenging to overrule an incorrect identity judgment than to make a correct identification independently. Moreover, the accuracy data for these trials further reflect that interference from the trial labels can facilitate both accuracy gains, as well as increases in error, compared to when a resolution was not provided by the labels.

In sum, this study shows that it is particularly difficult to accurately match faces when confronted with misleading identity information. Specifically, the reported experiments suggest that the commission of errors by automated systems is likely to undermine the performance of human observers, such as when an impostor is incorrectly labeled as an identity match. This has implications for human–computer interaction at passport control, where human operators verify the decisions of e‐Gates. The present results indicate that humans are unreliable at safeguarding against the errors of such systems.

## Supporting information


**Data S1.** Supplementary analysis of response times, *d*', and *criterion* across Experiments 1–3.

## Note

1 Note that this cross‐experiment comparison also produced a significant interaction of experiment and trial label, with the same pattern of significant effects, when this analysis compared accuracy for Block 3 only across experiments.
