# Supplementary material for: Human–Computer Interaction in Face Matching
Source: Cogn Sci. 2018 Jun 28;42(5):1714–32. doi: 10.1111/cogs.12633 (PMC6099365; doi:10.1111/cogs.12633)
Supplement: Supplementary file 1 — Data S1. Supplementary analysis of response times, d', and criterion across Experiments 1–3. [file COGS-42-1714-s001.docx]

**Supplementary Data for “Human-computer interaction in face matching”**

Matthew C. Fysh & Markus Bindemann

School of Psychology, University of Kent, Canterbury, UK

Correspondence to:

Matthew C. Fysh, School of Psychology, University of Kent, Canterbury CT2 7NP, UK.

Email: m.c.fysh@kent.ac.uk

Tel: +44 (0) 1227 827374

**Experiment 1**

*Response times*

Mean correct response times on consistent and inconsistent match trials were 3.90 and 4.87 seconds, respectively, and 4.31 seconds for unresolved trials. By contrast, response times were longer on mismatch trials, with 5.62 and 7.74 seconds for consistently-labelled and inconsistently-labelled trials, respectively, and 5.32 seconds when these were unresolved. A 2 (trial type) x 3 (trial label) within-subjects ANOVA revealed an effect of trial type, *F*(1,25) = 6.31, *p* < 0.05, η_p_^2^ = 0.02, due to faster responses on match trials. However, there was no effect of trial label, *F*(2,50) = 2.05, *p* = 0.14, η_p_^2^ = 0.08, and the interaction was not significant, *F*(2,50) = 0.57, *p* = 0.57, η_p_^2^ = 0.02.

*d’ and criterion*

For analysis of signal detection measures, the percentage accuracy data were converted to reflect overall sensitivity (*d’*) and response bias (*criterion*). For *d’*, a one-way ANOVA revealed an effect of trial label, *F*(2,58) = 3.79, *p* = 0.03, η_p_^2^ = 0.12. Paired-sample *t*-tests (with *alpha* corrected at *p* < 0.017) revealed that this was due to higher sensitivity on consistent versus unresolved trials, *t*(29) = 2.06, *p* < 0.05, and on inconsistent trials, *t*(29) = 2.72, *p* < 0.05. However, *d’* was comparable between inconsistent and unresolved trials, *t*(29) = 0.77, *p* = 0.45.

By contrast, the analogous analysis of *criterion* did not reveal a trial label effect, *F*(2,58) = 0.16, *p* = 0.85, η_p_^2^ = 0.01, due to near-identical scores of -0.19 for consistent and unresolved trial labels, and -0.14 on inconsistent trials.

**Experiment 2**

*Response times*

On match trials, mean correct response times increased from 3.21 seconds when trial labels were consistent, to 5.08 and 4.16 seconds when trial labels were inconsistent and unresolved, respectively. Response times on mismatch trials increased from 4.48 seconds when the trial labels were consistent, to 4.93 and 5.47 seconds when the labels were inconsistent and unresolved, respectively. These data reflect that responses were quickest when trial labels were consistent, but took longer when labels were misleading or did not resolve a given trial. However, a 2 (trial type) x 3 (trial label) within-subjects ANOVA did not reveal an effect of trial type, *F*(1,22) = 3.91, *p* = 0.06, ƞ_p_^2^ = 0.15, or an effect of trial label, *F*(2,44) = 1.48, *p* = 0.24, ƞ_p_^2^ = 0.06, and these factors did not interact, *F*(2,44) = 0.17, *p* = 0.84, ƞ_p_^2^ = 0.01.

*d-prime and criterion*

For *d’*, a one-way ANOVA revealed an effect of trial label, *F*(2,58) = 11.37, *p* < 0.001, ƞ_p_^2^ = 0.28. Paired-sample *t*-tests (with *alpha* corrected at *p* < 0.017) revealed that this was due to significantly lower *d’* on inconsistent trials, of 0.41, compared to 1.73 on consistent trials, *t*(29) = 3.64, *p* < 0.01, and 1.36 on unresolved trials, *t*(29) = 3.75, *p* < 0.01. However, sensitivity was comparable between consistent and unresolved trials, *t*(29) = 1.61, *p* = 0.12.

The analogous analysis of *criterion* also revealed an effect of trial label, *F*(2,58) = 3.63, *p* < 0.05, ƞ_p_^2^ = 0.11, with *criterion* shifting from -0.31 on inconsistent trials, to -0.25 and -0.09 on consistent and unresolved trials, respectively. Paired-sample t-tests (with *alpha* corrected at *p* < 0.017) showed that *criterion* was comparable between consistent and unresolved trials, *t*(29) = 2.00, *p* = 0.06, and between consistent and inconsistent trials, *t*(29) = 0.70, *p* = 0.49. However, *criterion* was significantly lower on inconsistent trials compared to unresolved trials, *t*(29) = 2.48, *p* < 0.05, suggesting that observers’ decisions were unfavourably guided by the labels when these provided misleading information.

**Experiment 3**

*Response times*

Due to an insufficient number of data points on unresolved trials between Blocks 1 and 2, only performance on consistent match and mismatch trials was analysed. For the first two blocks, a 2 (trial type) x 2 (block) within-subjects ANOVA did not reveal an effect of block, *F*(1,26) = 0.10, *p* = 0.75, ƞ_p_^2^ = 0.00, but of trial type, *F*(1,26) = 29.48, *p* < 0.001, ƞ_p_^2^ = 0.53, due to slower response times of 4.23 seconds on mismatch trials, compared to 2.59 seconds on match trials. The interaction was not significant, *F*(1,26) = 0.18, *p* = 0.67, ƞ_p_^2^ = 0.01.

The final block yielded insufficient data points for analysis, due to the low accuracy on inconsistent mismatch trials.

*d’ and criterion*

Between Blocks 1 and 2, *d’* increased slightly on consistent labels, from 1.94 to 2.24, respectively, as well as on unresolved trials, from 0.75 to 1.16. However, a 2 (trial label) x 2 (block) within-subjects ANOVA did not reveal an effect of block, *F*(1,29) = 2.20, *p* = 0.15, ƞ_p_^2^ = 0.07, but of trial label, *F*(1,29) = 19.39, *p* < 0.001, ƞ_p_^2^ = 0.40, due to superior performance on consistent trials. The interaction was not significant, *F*(1,29) = 0.07, *p* = 0.80, ƞ_p_^2^ = 0.00.

*Criterion* scores appeared comparable between Blocks 1 and 2 on consistent trial labels, with -0.50 and -0.45, respectively, as well as on unresolved trials, with -0.81 and -0.69, respectively. The 2 (trial label) x 2 (block) within-subjects ANOVA did not find an effect of trial label, *F*(1,29) = 0.52, *p* = 0.48, ƞ_p_^2^ = 0.02, but of block, *F*(1,29) = 5.21, *p* < 0.05, ƞ_p_^2^ = 0.15, due to a greater number of match responses in Block 2 compared to Block 1. These factors did not interact, *F*(1,29) = 0.09, *p* = 0.77, ƞ_p_^2^ = 0.00.

In Block 3, *d’* was at 1.95 when trial labels were consistent, but deteriorated to 0.68 and -0.26 on unresolved and inconsistent trial labels. A one-way ANOVA revealed that this trial label effect was reliable, *F*(2,58) = 15.14, *p* < 0.001, ƞ_p_^2^ = 0.34. Paired-sample *t*-tests (with *alpha* corrected at *p* < 0.017) demonstrated that this was due to significantly higher *d’* on consistent trials, compared to when these were inconsistent, *t*(29) = 5.49, *p* < 0.001, and unresolved, *t*(29) = 3.45, *p* < 0.01. In addition, *d’* was higher on unresolved trials than inconsistently-labelled trials, *t*(29) = 2.16, *p* < 0.05. The analogous analysis of *criterion* for Block 3 did not reveal an effect of trial label, *F*(2,58) = 0.68, *p* = 0.51, ƞ_p_^2^ = 0.02, with near-identical scores of -0.75 and -0.78 on inconsistent and unresolved trials, respectively, and -0.60 on consistent trial labels.
